# Modelling the causes of boiler accidents: implications for economic and social sustainability at the workplace^☆^

**DOI:** 10.1016/j.heliyon.2022.e09601

**Published:** 2022-06-07

**Authors:** Md. Ashiqur Rahman, Chitra Lekha Karmaker, Tazim Ahmed, Md. Ikram Khan, A.K.M Monjur Morshed, Syed Mithun Ali

**Affiliations:** a Department of Mechanical Engineering, Bangladesh University of Engineering and Technology, Dhaka 1000, Bangladesh; b Department of Industrial and Production Engineering, Jashore University of Science and Technology, Jashore 7408, Bangladesh; c Department of Industrial and Production Engineering, Bangladesh University of Engineering and Technology, Dhaka 1000, Bangladesh

**Keywords:** Boiler accidents, Neutrosophic AHP, Robust analysis, Emerging economy, Readymade garment industry

## Abstract

This study aims to examine the causes of boiler accidents in the context of the ready-made garment (RMG) industry of Bangladesh as an emerging economy. On the basis of a comprehensive review of the existing literature, previous accident reports, and technical discussion with relevant personnel in the industries and regulating authorities, a total of 14 causes of boiler accidents were identified. This study merged neutrosophic (N) theory with the analytic hierarchy process (AHP) for prioritizing the causes of boiler accidents. Finally, to examine the reliability of the results, a robustness analysis was performed. The findings reveal that the lack of standard legislation, non-standard boiler operation, use of expired, non-registered, and non-certified boilers, faulty design of boilers, and the shortage of skilled boiler operators are the top five notable causes of boiler accidents in the RMG industry. The findings provide valuable insights for industrial managers and policymakers to formulate strategies to reduce boiler accidents.

## 1 Introduction

Boiler is one of the common and indispensable components used by most of the industrial sectors in emerging economies (van der Gaast et al., 2007; Shen et al., 2017). The main purpose of using the boiler is to generate, store and supply steam as per requirement (Madejski and Żymełka, 2020). However, the failure of the industrial boiler may result in catastrophic accidents which eventually causes huge losses to the assets and personnel (Ahmed and Gu, 2020). In Bangladesh, boilers are widely used in many industries for various purposes in different stages of their operation. Among these industries, the ready-made garment (RMG) industry, one of the leading remittance-earning industries of Bangladesh, uses boilers mainly to dry clothes and provide steam for ironing (Patil and Bewoor, 2020).

The RMG industry is crucial to the socio-economic development such as poverty mitigation and women empowerment in Bangladesh (Sarkar et al., 2020), and this industry generated more than 84% of the country's export earnings in the fiscal year 2018–2019, with a worth of approximately 34.13 billion USD (Ali and Habibullah, 2019). Despite the significant contribution of the RMG industry to the socio-economic development of Bangladesh, ensuring the occupational health and safety (OHS) at the workplace in this industry has become a great challenge. This industry has been plagued by several accidents such as structural collapse of the factory buildings, catastrophic boiler blasts, massive fire incidents, and fatal boiler accidents (Barua and Ansary, 2017; Rahim, 2017; Claeson, 2015). In the last few years, a significant number of boiler accidents have been observed in Bangladesh, many of them being catastrophic (Chakraborty et al., 2020). Two deadly boiler accidents in Multifabs Ltd. apparel and Tampaco Foils Ltd., caused the death of around 40 people with serious injuries to about 100 workers (Akhter et al., 2019; Rahim, 2017). A garment factory near Dhaka faced a serious boiler accident with the death of 1 worker and injuries of several people (“Bangladesh: Boiler Explosion”, 2019). Recently, another boiler explosion has been occurred at a rice mill in Habiganj where 1 worker was killed and 5 workers were seriously injured (“One Killed, Five Hurt”, 2020). In the northern part of Bangladesh, a boiler accident was occurred due to boiler explosion and caused the death of 1 worker (“Man Killed, 9 Hurt”, 2020). According to all these reports, it is evident that there are frequent fatal boiler accidents in Bangladesh.

The consequence of boiler accidents in the RMG industry is catastrophic as these accidents not only cause substantial loss of property and lives, but also severely affect the progress of socio-economic development by disrupting the sustainable operations of that industry (Barua and Ansary, 2017; Paul et al., 2020; Rahim, 2017). Such accidents also cause a loss of reputation of the RMG industry at the global apparel market. In order to sustain the global competition, ensuring a safe and healthy working environment is pivotal (Hald, 2018). The abovementioned accidents have raised the important issues of ensuring safe boiler operation in Bangladesh on an urgent basis. Thus, evaluating and modelling the causes of boiler accidents in the RMG industry is crucial to industrial managers and government policymakers.

Over the last few years, some researchers have attempted to identify the causes of boiler accidents and provided guidelines of safe boiler operations (Park et al., 2020; Pastor et al., 2020; Deghal et al., 2019; Trojan, 2019; Hong et al., 2019; Huang et al., 2019). Park et al. (2020) analyzed the characteristics of 203 bio liquid samples as a substitute fuel for safe boiler operation. The results revealed that bio liquid is reliable and safe for fire tube boilers in terms of low emissions and reduced boiler accidents. Pástor et al. (2020) used quantification of residual stresses to identify the possible causes of the boiler's pipe failure. The findings depicted that rather than temperature change, high levels of residual stress in the piping system of the steam boiler is the most vital cause for pipe failure. Deghal et al. (2019) stressed on maintaining optimum operating condition to reduce the probability of boiler accidents. Trojan (2019) emphasized the selection of correct parameters for safe boiler operations and developed a non-linear mathematical model for identifying the correct values of these parameters through simulation. Some studies have focused on the improvement of control systems for ensuring the boiler safety and proper operations (Hong et al., 2019; Huang et al., 2019). Ahmed and Gu (2020) used an accident-based Failure Modes, Effects and Criticality Analysis (FMECA) to sort out the potential failure modes of the marine boilers and the prioritization of the risks were done using fuzzy expert systems.

Moreover, faulty design of the piping system as well as other parts of the boiler system is responsible for the unsafe boiler operations and accidents (Cholette et al., 2019). Kumar et al. (2018) found that the hot corrosion of boiler tubes and erosion are primarily responsible for early boiler failure which may lead to unsafe boiler operations and accidents. Improper control system increases the possibility of boiler accidents and also affects the energy consumption rate (Barma et al., 2017). Duarte et al. (2017) analyzed the failure of boilers through residual stress measurements, chemical analysis and microstructure analysis and identified that the quality of the feed water was one of the main reasons for early boiler failure and accidents. Ai et al. (2015) applied the fault tree analysis method based on safety ergonomics theories to sort out the key factors causing boiler water shortage accidents. Rahmani et al. (2009) used a multipurpose best estimate (BE) thermal-hydraulic system code to assess the causes of boiler tubes’ overheating mechanisms and identified the loss of feed water as one of the causes of boiler accidents.

Even though several possible causes of boiler accidents have been identified by several studies, most of them are analyzed in isolation. To formulate effective mitigation strategies to boiler accidents, industrial managers need to evaluate all possible causes of the accidents thoroughly. However, such type of evaluation is not easy as it is a complex multi-criteria decision-making process because of having inadequate information and insufficient germane literature (Ng et al., 2002; Papadopoulos et al., 2010; Manu et al., 2018; Ardy et al., 2021).

A few studies appear in the literature on the boiler safety of the RMG industry of emerging economies. Paul et al. (2020) conducted a study on the boiler safety in the context of emerging economies in South Asia and identified several causes such as non-compliance of standards, lack of safety awareness, faulty design etc. for boiler accidents. Ali and Habibullah (2019) examined the overall boiler safety scenario in Bangladesh and found that most of the industries have ignored the safety issues of boiler as they were using un-registered boilers.

The above discussion reveals that modelling of possible causes of boiler accidents in the context of the RMG industry of emerging economy is absent in the literature. Therefore, for bridging the aforesaid research gaps, this study aims to identify, analyze, and evaluate the causes of boiler accidents in the RMG industry of Bangladesh as an emerging economy.

To fulfill the aims, first, the causes of boiler accidents were identified by comprehensive review of the existing literature, analyzing previous accident reports, and technical discussion with relevant personnel in the industries and regulating authorities. Then, to examine and analyze the possible causes, this study merged the neutrosophic (N) theory with one of the multi-criteria decision-making (MCDM) methods namely the analytic hierarchy process (AHP). This study has applied the AHP because of the advantage of this method of analyzing the causes based on the cognitive knowledge of the experts. Besides, integration of neutrosophic theory deals with the incompleteness, inconsistency and the lack of determinacy of the information, which leads to the robustness of the neutrosophic analytic hierarchy process model. Finally, to examine the reliability of the results, a robustness analysis was performed.

This paper is structured as follows. Section 2 presents the methodological framework which is followed by results in Section 3. The discussion part of the obtained results along with implications is outlined in Section 4. Finally, Section 5 concludes the research with limitations and scopes for further analysis.

## 2 Methodology

**Figure 1:**
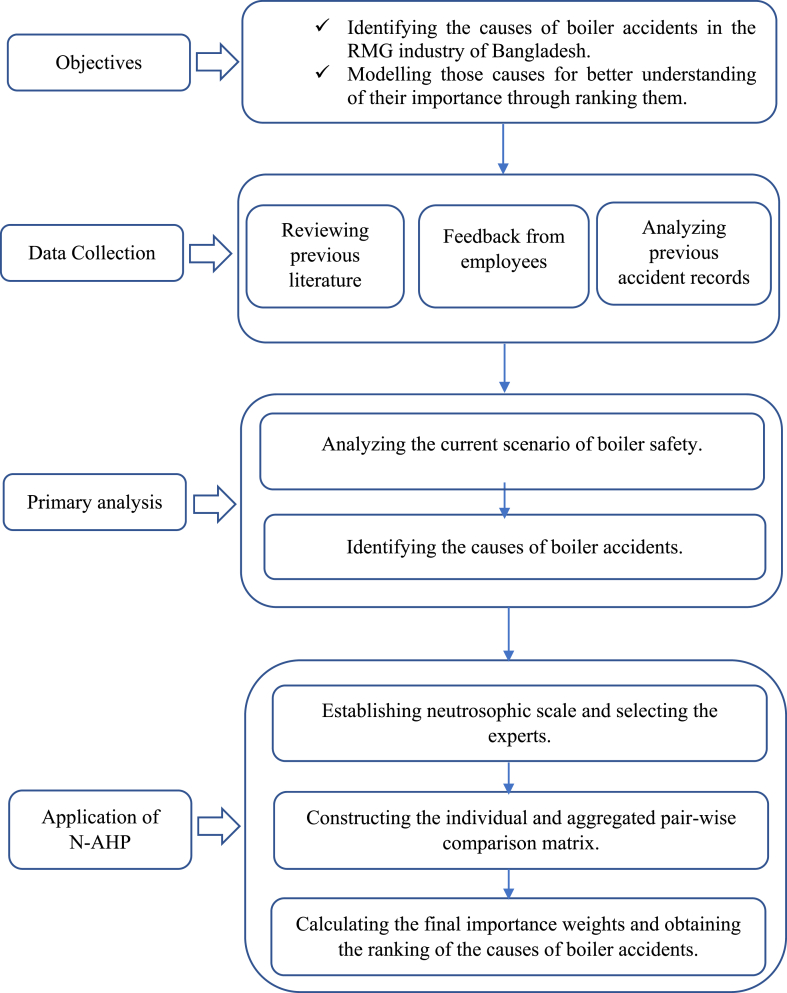
Proposed methodological framework.

Figure 1 presents the methodology proposed in this research. Other researchers can replicate the steps used in this study.

### 2.1 Context and data

The current study has intended to explore and analyze the causes of boiler accidents in the RMG industry of Bangladesh as an emerging economy. The RMG industry was selected as the case study because more than 60% of the existing boilers in Bangladesh are being utilized by this leading industry (Khan et al., 2018). Before undertaking the remedial actions, all the possible causes of boiler accidents need to be explored and examined systematically. However, to identify the causes of boiler accidents, the current scenario of the existing operational boilers in the RMG industry of Bangladesh is required to be analyzed. Therefore, at the first step of the study, the information available on the currently operational boilers from the RMG industry of Bangladesh were collected for analyzing the boiler safety scenario. The data were collected from the regulating and enforcing agency in Bangladesh called the Office of the Chief Inspector of Boilers (CIB). The findings revealed that although there are about 5,000 registered boilers currently operating in Bangladesh, the total number of boilers operating in the country, according to the estimate of the Office of the CIB, is more than 10,000.

In the next step, the study incorporates the feedback from the personnel working in the RMG industry of Bangladesh on the existing boiler safety scenario. A total number of 40 employees including boiler operators, technicians, engineers, and compliance managers were selected for eliciting the feedback after stating the objectives of this study. A workshop on boiler safety was conducted with these employees at Bangladesh University of Engineering and Technology (BUET), Bangladesh. The participants were asked to answer the questions: a) *What are the possible causes of boiler accidents in the RMG industry? b) How can the existing boiler safety scenario be improved?* The feedback was recorded manually to identify the causes of boiler accidents in the RMG industry of Bangladesh. Then, these causes have been analyzed further using neutrosophic analytic hierarchy process (AHP) based on experts' opinion. Since there are inadequate data regarding the loss of life, injury, and economic consequences, the current study has relied on experts’ experiences and knowledge to rank the causes of boiler accidents. To better interpret the opinion of experts, the current study has adopted neutrosophic AHP method that can provide reliable output under insufficient data (Vafadarnikjoo et al., 2021; Darko et al., 2018).

### 2.2 Neutrosophic theory

Smarandache introduced the neutrosophic logic in 1995 to deal with the vagueness and incompleteness of elicited information (Rivieccio, 2008). Neutrosophic sets are developed based on the classical sets, fuzzy sets and intuitionistic fuzzy sets (Smith, 2019). Fuzzy sets are used to express the imprecise or incomplete information with the degree of membership only. Fuzzy sets were advanced by combining with the intuitionistic sets, introduced by Liu and Yuan (Liu, 2014; Mahmood et al., 2018), to express the incompleteness more specifically with degree of membership and degree of non-membership values. However, the lack of consistency and determinacy within the information cannot be handled by the intuitionistic fuzzy sets. Therefore, trapezoidal neutrosophic fuzzy set (TNFS) was introduced by Ye (2015) to handle the inconsistency, indeterminacy and incompleteness of information. These are expressed and handled through truth-membership (TM), indeterminacy-membership (IM) and falsity-membership (FM) functions, respectively. The theory of TNFS is based on the trapezoidal fuzzy sets and single valued neutrosophic set (Broumi et al., 2019). The next section details some basic concepts pertaining to TNFS, their operations and aggregation operator.

*Definition 1*: (Khatter, 2020) Suppose K is defined as a universe and discourse and $\tilde{A}$ is defined as a TNFS in K. Then, the single valued TNFS $\tilde{A}$ can be expressed as follows.

(1) $$\tilde{A}=\left\{<k,\;T_{\tilde{A}}\left(k\right),\;I_{\tilde{A}}\left(k\right),\;F_{\tilde{A}}\left(k\right)>|k\in K\right\},$$

Where, $T_{\tilde{A}}\left(k\right)$, $I_{\tilde{A}}\left(k\right)$ and $F_{\tilde{A}}\left(k\right)$ denote the TM, the IM and the FM function respectively. $T_{\tilde{A}}\left(k\right)\subset \left[0,\;1\right]$, $I_{\tilde{A}}\left(k\right)\subset \left[0,\;1\right]$ and $F_{\tilde{A}}\left(k\right)\subset \left[0,\;1\right]$ are three trapezoidal fuzzy numbers $T_{\tilde{A}}\left(k\right)=\left(t_{\tilde{A}}^{1}\left(k\right),t_{\tilde{A}}^{2}\left(k\right),\;t_{\tilde{A}}^{3}\left(k\right),t_{\tilde{A}}^{4}\left(k\right)\right)\;:K\to \;\left[0,\;1\right]$; $I_{\tilde{A}}\left(k\right)=\left(i_{\tilde{A}}^{1}\left(k\right),i_{\tilde{A}}^{2}\left(k\right),\;i_{\tilde{A}}^{3}\left(k\right),i_{\tilde{A}}^{4}\left(k\right)\right)\;:K\to \;\left[0,\;1\right]$ and $F_{\tilde{A}}\left(k\right)=\left(f_{\tilde{A}}^{1}\left(k\right),f_{\tilde{A}}^{2}\left(k\right),\;f_{\tilde{A}}^{3}\left(k\right),f_{\tilde{A}}^{4}\left(k\right)\right)\;:K\to \;\left[0,\;1\right]$ with the condition $0\leq t_{\tilde{A}}^{4}\left(k\right)+i_{\tilde{A}}^{4}\left(k\right)+f_{\tilde{A}}^{4}\left(k\right)\leq 3$.

*Definition 2*: (Ye, 2015) Trapezoidal neutrosophic number weighted arithmetic averaging (TNNWAA) operator. Let ${\tilde{k}}_{j}=<\left(a_{j},b_{j},c_{j},d_{j}\right),\left(l_{j},m_{j},n_{j},o_{j}\right),\left(p_{j},q_{j},r_{j},s_{j}\right)>$ ; (j=1,2,3,...,k) be the collection of trapezoidal neutrosophic numbers (TNN). Then the aggregation operator TNNWAA can be defined as follows:

(2) $$TNNWAA\left({\tilde{k}}_{1},\;{\tilde{k}}_{2},\;...,\;{\tilde{k}}_{n}\right)=\;w_{1}{\tilde{k}}_{1}⊕w_{2}{\tilde{k}}_{2}⊕\;...\;⊕w_{k}{\tilde{k}}_{k}={{⊕}_{j=1}}^{k}\left(w_{j}{\tilde{k}}_{j}\right)$$

Where $w_{j}\left(j=\;1,\;2,\;...,\;k\;\right)$ is the weight of the j^th^ TNN ${\tilde{k}}_{j}\left(j\;=\;1,\;2,\;...,\;k\right)$ with $w_{j}\in \;\left[0,\;1\right]$ and $\overset{k}{\underset{j=1}{\sum }}w_{j}=1$. The aggregated value using the TNNWAA operator is also a TNN and can be written as follows:

(3) $$TNNWAA\left({\tilde{k}}_{1},{\tilde{k}}_{2},...,{\tilde{k}}_{k}\right)=w_{1}{\tilde{k}}_{1}⊕w_{2}{\tilde{k}}_{2}⊕...⊕w_{k}{\tilde{k}}_{k}=\;{{⊕}_{j=1}}^{k}\left(w_{j}{\tilde{k}}_{j}\right)=<\left(1-\overset{k}{\underset{j=1}{\prod }}{\left(1-a_{j}\right)}^{w_{j}},1-\overset{k}{\underset{j=1}{\prod }}{\left(1-b_{j}\right)}^{w_{j}},\;1-\overset{k}{\underset{j=1}{\prod }}{\left(1-c_{j}\right)}^{w_{j}},1-\overset{k}{\underset{j=1}{\prod }}{\left(1-d_{j}\right)}^{w_{j}}\right),\;\left(\overset{k}{\underset{j=1}{\prod }}l_{j}^{w_{j}},\overset{k}{\underset{j=1}{\prod }}m_{j}^{w_{j}},\overset{k}{\underset{j=1}{\prod }}n_{j}^{w_{j}},\overset{k}{\underset{j=1}{\prod }}o_{j}^{w_{j}}\right),\;\left(\overset{k}{\underset{j=1}{\prod }}p_{j}^{w_{j}},\overset{k}{\underset{j=1}{\prod }}q_{j}^{w_{j}},\overset{k}{\underset{j=1}{\prod }}r_{j}^{w_{j}},\overset{k}{\underset{j=1}{\prod }}s_{j}^{w_{j}}\right)\;>$$

### 2.3 Neutrosophic analytic hierarchy process (N-AHP)

Analytic hierarchy process (AHP), a very popular type of multi-criteria decision making (MCDM) methods, was introduced by Thomas Saaty (1990). This method has widely been used in complex decision-making problems in the area of safety and risk analysis (Li et al., 2020; Kabo-bah et al., 2021), maintenance management (Pagano et al., 2021), supply chain management (Mastrocinque et al., 2020), project management (Büyüközkan et al., 2021) etc. The main advantage of this method is that the alternatives for the complex decision-making process are analyzed by the experts from the mathematical and psychological point of view. However, the AHP method involves the experts’ opinions for ranking the alternatives or factors based on linguistic judgments and therefore, there exists some uncertainty and incompleteness in the elicited information (Javed et al., 2020). Sometimes multiple decision makers or experts are involved in the AHP and hence, inconsistency becomes an issue while developing the pairwise comparison matrix. This study has intended to identify and model the causes of boiler accidents in the RMG industry which is a complex decision-making process because of having not enough information and insufficient literature on it. Considering all these shortcomings, the current study has hybridized AHP with trapezoidal neutrosophic set to handle the uncertainty and incompleteness of information as well as the inconsistency in the judgments. The computational steps of this hybrid N-AHP method have been described as follows.

*Step 1*. Defining the objectives and identifying the alternatives.

The objectives of the current study are to identify, analyze and model the possible causes of boiler accidents in the RMG industry. The possible causes of boiler accidents have been considered as the alternatives for evaluation.

*Step 2. Selecting the experts* and *establishing the neutrosophic scale.*

The analysis and modelling the causes of boiler accidents in the RMG industry require substantial knowledge and experiences of the experts regarding the operations and other important variables of boiler. Moreover, N-AHP can provide consistent and reliable results with relatively small sample size provided that the respondents are expert in the respective field (Darko et al., 2018). Therefore, the current study has selected 10 experts who have great knowledge and more than 15 years of experience in boiler operation in various RMG companies based on the purposive sampling method. Purposive sampling method is used when to select respondents from a specific domain of interest (White et al., 2018). These 10 experts were chosen for having knowledge in the boiler technology domain. However, the working experiences of these experts were not the same. In this case, the weights of the experts were given based on the year of their working experiences and hence, a weight of 0.15, 0.15, 0.15, 0.10, 0.10, 0.10, 0.10, 0.05, 0.05, 0.05 were assumed to Expert 1 to Expert 10, respectively. The sum of these weights is 1. The profile of these experts with their year of working experience are given in Table 1.

**Table 1:** Profile and working experience of the experts.

Experts	Designations	Experiences
Expert 1	General Manager	20 years
Expert 2	Chief Operating Officer	19 years
Expert 3	Chief Operating Officer	20 years
Expert 4	Production Manager	15 years
Expert 5	Production Manager	14 years
Expert 6	Production Manager	16 years
Expert 7	Production Head	15 years
Expert 8	Production Officer	10 years
Expert 9	Deputy Manager	8 years
Expert 10	Manager	11 years

The neutrosophic scale is also constructed in this step using Eq. (1) and Eq. (2) based on which the experts make their judgements to give the preference of one alternative over another. Table 2 shows the neutrosophic scale used in the current study that contains the single value trapezoidal neutrosophic number (SVTNN) for the corresponding linguistic term.

**Table 2:** The neutrosophic scale in single value trapezoidal neutrosophic number (SVTNN).

Numerical scale	Linguistic scale	Trapezoidal neutrosophic scale
1	Equal importance	<(1,1,1,1);0.5,0.5,0.5>
3	Moderate importance	<(2,3,6,7);0.3,0.75,0.7>
5	Strong importance	<(4,5,6,7);0.8,0.15,0.2>
7	Very strong importance	<(6,7,8,9);0.9,0.1,0.1>
9	Extreme importance	<(9,9,9,9);1,0,0>
2,4,6,8	Intermediate values	

*Step 3.* Constructing *the pairwise comparison matrix.*

In this step, a pairwise comparison matrix is developed based on the opinions from the experts. The experts provide their opinions using the linguistic scale from Table 2.

*Step 4. Constructing* the *aggregated pair-wise comparison matrix.*

Aggregation of the individual pair-wise comparison matrix is performed using the TNNWAA operator. Eq. (3) is used to perform the TNNWAA for developing the pair-wise comparison matrix.

*Step 5. Determining the* neutrosophic *synthetic values.*

The following equation is used to determine the neutrosophic synthetic value $S_{i}$ for each element.

(4) $$S_{i}=\;\overset{k}{\underset{j=1}{\sum }}\rho_{ij}\times {\left[\overset{n}{\underset{i=1}{\sum }}\overset{n}{\underset{j=1}{\sum }}\rho_{ij}\right]}^{-1},\;i=1,2,\;...\;,$$

Where, n is the number of elements and $\rho_{ij}$ is the (i,j)^th^ element of the aggregated neutrosophic pair-wise comparison matrix in SVTNNs.

*Step 6*. Calculating the final importance weights.

The final importance weights $W_{i}$ of the alternatives are calculated using the Eq. (5) and the weights are in SVTNNs. However, these SVTNNs are required to convert into crisp values for comparing the weights. The crisp values $C_{i}$ are obtained using the score function and Eq. (6).

(5) $$W_{i}=\frac{S_{i}}{\sum_{i=1}^{k}S_{i}}\;i=1,\;2,\ldots ,\;k.$$

(6) $$C_{i}\;=\;\left(\frac{1}{12}\right)\times \left(\alpha_{i}+\;\beta_{i}+\;\gamma_{i}+\delta_{i}\right)\times \left(2+\varphi_{i}-\theta_{i}-\psi_{i}\right)\;i=1,\;2,\;\ldots ,\;k.$$

Where, $<\left(\alpha_{i},\;\beta_{i},\;\gamma_{i},\;\delta_{i}\right);\;\varphi_{i},\;\theta_{i},\;\psi_{i}>$ is the final importance weight of i^th^ alternative.

## 3 Results

### 3.1 Identification of causes of boiler accidents

As per the *Boiler Act of Bangladesh (1923)*, every boiler accident should be reported to the office of the chief inspector of boilers (CIB) and license of that specific boiler must be renewed before it can resume operation. However, in practice, the accidents which are not catastrophic or highly publicized are rarely reported. The reports of the official investigation committee, formed by the office of the CIB, investigating ten major boiler accidents took place in the last eight years have been collected and analyzed. In the next step, a survey was conducted at Bangladesh University of Engineering and Technology (BUET) among the 40 participants working in various RMGs of Bangladesh who have the responsibilities to manage the boiler operations. These 40 participants were chosen based on purposing sampling method (White et al., 2018). The purpose of the survey was to explore the causes of boiler accidents in the RMG industry of Bangladesh from the employees' point of views. A brief description of the profile of these 40 participants has been given in Table A1 (Appendix A). After aggregating all the information from the survey feedback, previous boiler accidents’ investigation reports, and reviewing the previous literature, a total of 14 possible causes of boiler accidents in the RMG industry of Bangladesh were identified as shown in Table 3 with relevant sources. These 14 causes were further analyzed to determine their importance weights using the N-AHP approach.

**Table 3:** List of possible causes of boiler accidents in the RMG industry.

Code	Causes of boiler accidents	Sources
C1	Lack of regular boiler inspection and maintenance	Survey + Investigation record
C2	Use of expired, non-registered, and non-certified boilers	Investigation record
C3	Non-standard boiler operation	Investigation record
C4	Faulty design of boiler	Literature review (Cholette et al., 2019)
C5	Improper control and safety system of boiler	Literature review (Barma et al., 2017)
C6	Corrosion of critical parts of the boiler	Literature review (Kumar et al., 2018)
C7	Low quality material for boiler manufacturing	Investigation record
C8	Lack of standard legislation	Investigation record
C9	Lack of standard occupational health and safety practices	Investigation record + Literature review (Khan et al., 2018)
C10	Shortage of skilled boiler operators	Survey + Investigation record
C11	Inadequate regulatory authority	Survey
C12	Failure of the safety valve	Investigation record
C13	Poor water treatment	Literature review (Duarte et al., 2017)
C14	Conditions of the boiler room	Investigation record

### 3.2 N-AHP results

In the current study, the objectives were to identify the causes of boiler accidents in the RMG industry and rank those causes. The identified 14 causes of boiler accidents, represented in Table 3, were considered as the decision-making alternatives for evaluation in N-AHP. After that the list of possible causes of boiler accidents were sent to the previously selected 10 experts (Table 1) and they were requested to give the preference of one cause over another with the help of linguistic terms according to Table 2. The inputs from the experts have been given in Table B3 – B12 (Appendix B). Based on the feedbacks accumulated from the experts, the individual pairwise comparison matrix was developed. Total 10 pairwise comparison matrices were formed. All the pairwise comparison matrices were then aggregated using the TNNWAA operator according to Eq. (3). Table B13 (Appendix B) presents the aggregated pair-wise comparison matrix obtained by aggregating the individual matrix from the experts. The neutrosophic synthetic value of each cause was calculated from the aggregated pair-wise comparison matrix using Eq. (4). Table B14 (Appendix B) shows the neutrosophic synthetic value of each cause. The final importance weights in SVTNNs of each cause was calculated from the neutrosophic synthetic values using the Eq. (5) and Eq. (6). Table B15 (Appendix B) shows the final importance weights of the causes in SVTNNs. For better understanding the ranking of importance of the causes, these SVTNNs were converted into crisp values using the Eq. (6). Table 4 represents the final crisp weights of the causes with the ranking. It has been found that “Lack of standard legislation (C8)” is the most important cause of boiler accidents in the RMG industry. The final crisp weight of this cause is 0.153. “Non-standard boiler operation (C3)” and “Use of expired, non-registered, and non-certified boilers (C2)” are the second and third most important causes of boiler accidents and their weights are 0.137 and 0.122, respectively. The least important cause is found to be “Conditions of the boiler room (C14)” as the weight of this cause is 0.009.

**Table 4:** Final importance weights of the causes of boiler accidents.

Causes	Code	Weight	Rank
Lack of regular boiler inspection and maintenance	C1	0.051	9
Use of expired, non-registered, and non-certified boilers	C2	0.122	3
Non-standard boiler operation	C3	0.137	2
Faulty design of boiler	C4	0.109	4
Improper control and safety system of boiler	C5	0.069	7
Corrosion of critical parts of the boiler	C6	0.028	11
Low quality material for boiler manufacturing	C7	0.023	12
Lack of standard legislation	C8	0.153	1
Lack of standard occupational health and safety practices	C9	0.067	8
Shortage of skilled boiler operators	C10	0.098	5
Inadequate regulatory authority	C11	0.086	6
Failure of the safety valve	C12	0.038	10
Poor water treatment	C13	0.012	13
Conditions of the boiler room	C14	0.009	14

## 4 Discussions and implications

The current study has intended to explore and analyze the causes of boiler accidents in the RMG industry of Bangladesh for better understanding the comparative significance of the causes. Table 4 has shown that *“Lack of standard legislation (C8)”* is the most critical cause of boiler accidents in the RMG industry of Bangladesh. Boiler systems and operations must follow some local and international legislations for ensuring the operational safety of existing boilers and proper maintenance activities. The national legislation and standards germane to the permit, installation, and operation of boilers and ensuring occupational safety in Bangladesh are ‘*The Boiler Act, 1923’*, ‘*Bangladesh Boiler Regulation-1951*’, *‘Bangladesh National Building Code, 2020’* and ‘*The Bangladesh Labour Act, 2006’*. However, these acts and regulations do not provide a detailed guideline on the proper maintenance and operations of boilers. The BNBC, 2020 has some basic guidelines on boiler safety especially on boiler installation and requirements of the boiler room. But it also has some lacking like testing, inspection and maintenance that need to be improved to ensure better safety for boiler operation. This N-AHP result has also revealed that *“Non-standard boiler operation (C3)”* is the second most influential cause of boiler accidents in the RMG industry in the context of Bangladesh. The standard operating procedures of the boiler operation is often not followed, and the boiler operation does not comply with the health and safety regulations. The non-standard boiler operation results from a combination of factors such as lack of specific directives in the relevant legislations, poor safety culture, lack of awareness on the management and owner's part, technical inadequacy of the operating and maintenance personnel, and so on. Ali and Habibullah (2019), in their study, also reported that following the standard regulation and legislation can reduce the possibility of boiler accidents significantly.

Underlying investigation of the current study has found that many boilers in the RMG industry of Bangladesh continue to be used even when their lifetime and licenses have expired. From Table 4, it can also be seen that *“Use of expired, non-registered, and non-certified boilers (C2)”* has found to be the third most influential cause of boiler accidents. It can be noted here that in case of the two most deadly boiler accidents in Bangladesh in the recent times (Multifabs Ltd. and Tampaco Foils Ltd.), the use of expired or unauthorized boilers was reported to be one of the major reasons. In the case of Multifabs Ltd., the boiler was being operated even after its date of expiration (The Daily mail, July 5, 2017). In the case of the boiler accident at Tampaco Foils Ltd. in 2016, it was reported that the boiler being used was unauthorized. On the other hand, *“Faulty design of boiler (C4)”* has found to be the fourth most influential cause of boiler accidents, since sometimes, accidents occur due to the faults in design of the boiler system. Therefore, boiler system design should include the early fault detection mechanism for avoiding such accidents (Khan et al., 2018). However, most of the boilers of the RMG industry do not have any early fault detection mechanism incorporated with the design of the boiler.

Moreover, there is a shortage of skilled and certified boiler operators in Bangladesh. Safe boiler operations largely depend on the competency and skill of the personnel operating and maintaining the boilers. The investigation records of the previous boiler accidents in Bangladesh have also shown that in most of the cases, the boiler operators were uncertified or had very little or no training. *“Shortage of skilled boiler operators (C10)”* has been ranked as the fifth most influential cause of boiler accidents in the RMG industry. Continued boiler operations by the uncertified and unqualified boiler operators has been suggested by the official reports as a major reason behind the fatal boiler accidents of the recent past.

Many boiler accidents have occurred in the RMG industry due to *“Lack of standard occupational health and safety practices (C9)”*. The lack of occupational safety guidelines and the lack of proper regulations on boiler operations often result in an inadequate culture of testing, maintaining and monitoring of boilers. The boiler owners and the industry management are the responsible authority to ensure the required occupational safety and health and standard associated with the whole boiler system. *“Lack of regular boiler inspection and maintenance (C1)”* is another primary cause of boiler accident in the RMG industry of Bangladesh. However, most of the cases, the industry management ignores the necessity of carrying regular inspection and periodic maintenance of the boiler system. They focus on preventive and corrective maintenance instead. Proper documentation, inspection reports, and log-books are not maintained by the management for the inspection and maintenance. Besides, the underlying investigation of the previous boiler accidents records has also revealed that one of the common causes of the accidents was the *“Lack of regular boiler inspection and maintenance (C1)”*.

Some technical reasons are also found to be associated with the boiler accidents in the RMG industry of Bangladesh. *“Failure of the safety valve (C12)”* is observed to be one of the common technical causes of boiler accidents. Technical factors like *“Corrosion of critical parts of the boiler (C6)”* and *“Low quality material for boiler manufacturing (C7)”* have been marked as two common causes of boiler accidents. Boiler accidents have also been reported due to the *“Poor water treatment (C13)”* and *“Conditions of the boiler room (C14)”* - but these factors were less influential than the other ones.

### 4.1 Implications for management and policymakers

To ensure the proper boiler safety, the boiler owners, users and the management need to give attention to develop some preventive measures and some strategies. There is a lack of understanding on the boiler owners' part that the owner or user of a boiler is ultimately responsible for ensuring that the boiler system complies with all the relevant health and safety regulations. The development of the appropriate risk assessment framework and carrying out the program, identifying all possible sources of danger and operational risks, level of supervision and maintenance, etc. need to be properly prioritized on an urgent basis (Cicek and Celik, 2013; Khan and Abbasi, 2001). Based on the outcomes of the study, the employees, professionals and RMG owners can formulate strategic decisions to improve the boiler safety through optimal usage of their resources. The policymakers and the industry management can use the proposed N-AHP model to make further analysis through adding more causes of boiler accidents relevant to their industries. The proposed N-AHP model has the flexibility to add more expert's opinions for further analysis.

The RMG owners and management, along with all the relevant stakeholders, such as the policymakers, the regulating authorities. International bodies such as International labor Organization (ILO), international retailer brands and such, must focus on the main causes of boiler accidents outlined in the present study. For example, to address the most influencing reason identified behind boiler accidents - *“Lack of standard legislation (C8)”*- the RMG owners and all the concerned authorities must work together to introduce significant amendment of the existing legislations and enact new regulations with adequate provisions to ensure boiler safety. They should also focus on implementing carefully drafted boiler safety measures in all the RMG factories and enforcing punishments for any violation of the laws. Establishing standard legislation and ensuring the proper implementation, this legislation will also assist the RMG industry to improve their business performances. The industry owners and manager also need to address the issue of *“Non-standard boiler operation (C3)”* adequately. To do that, they must emphasize on raising the OHS awareness among its workers and employees. By implementing a robust safety ethics, a culture of compliance, and promoting adherence to the standard operational procedures and practices, they can adequately address the ‘non-standard’ issue of their boiler operation. The employees also need to be careful to comply with the expiry date, registration and proper certification of boilers. As a large number of boiler accidents are due to lack of proper maintenance, regular inspection, and testing of the boiler system, the management must ensure a more dynamic and regular inspection, testing and maintenance regime. Implementation of these interventions will reduce the possibilities of boiler accidents significantly. Another responsibility of the owner and the employees is to arrange adequate training for the boiler operators and maintenance personnel on standard operations. They can also work with the policymakers and licensing authorities to ensure that there is a larger pool of licensed and certified boiler operators. Finally, the outcomes of this study will help the owners and the employees to rethink about the overall safety system of boilers. The ranking of the causes will help them to allocate the resources wisely while taking actions to improve the boiler safety.

### 4.2 Implications for sustainable development goals (SDGs)

The proposed N-AHP model for boiler accidents in the RMG industry, will assist policymakers to formulate mitigating strategies for achieving some of SDGs in Bangladesh as an emerging economy. According to the model, “Lack of standard legislation”, “Non-standard boiler operation”, “Use of expired, non-registered, and non-certified boilers”, “Faulty design of boiler” are the top most causes responsible for boiler accidents in the RMG industry of Bangladesh, an emerging economy. Address the above-mentioned causes will improve the legislative measures and collaboration among all parties at the national and international forum towards sustainability. As a result, Partnerships for the Goals (SDG 17) can be ensured through focusing on the above-mentioned causes. “Lack of standard occupational health and safety practices (C9)” will not only force the industry management to ensure the required safety during the boiler operation, but it will also promote the sound and healthy working environment for the workers of the RMG industry. This activity eventually will help the policymakers regarding the achievement of Good Health and Well-Being (SDG 3). Moreover, achievement of Decent Work and Economic Growth (SDG 8) can be expedited through ensuring the standard occupational health and safety practices.

Upon identifying the possible causes of boiler accidents, the industry owners should develop mitigating strategies to reduce the possibilities of boiler accidents. Through implementing standard rules and regulations, continuous monitoring of the boiler operation, and ensuring skilled operators, the number of boiler accidents will be reduced which ultimately will ensure a sustainable industry, Innovation and Infrastructure (SDG 9). Moreover, the use of boilers is rapidly increasing in the thermal and nuclear power plants of Bangladesh with the ultimate goal transforming the country into a developed one. Therefore, to ensure the sustainable and affordable energy source to all communities (SDG 7), safe operation of boilers is very crucial. Thus, the proposed N-AHP model is expected to contribute to Good Health and Well-Being (SDG 3); Affordable and Clean Energy (SDG 7); Decent Work and Economic Growth (SDG 8); Industry, Innovation and Infrastructure (SDG 9) via resilient strategies.

### 4.3 Robustness analysis of the results

The ranking of the “causes of boiler accidents” has been obtained from the judgments of the experts using N-AHP. Therefore, it is necessary to examine the reliability of the results through robust analysis (Ivanco et al., 2017; Ram and Chandna, 2018). Here, the robustness analysis has been carried out by varying the weights of experts (Seker and Zavadskas, 2017; Karmaker and Ahmed, 2020). The weight distributions were constructed for 5 scenarios and N-AHP has been performed for each scenario. The results of these analyses have been shown in Table 5. The first scenario represents the weights of the experts that have been used in the actual analysis. In the second scenario, all the experts were given the equal weight (0.10). After that, the weights of the experts have been varied randomly for the next 3 scenarios; however, the weights were kept higher for the more experienced experts whereas the weights were kept lower for the less experienced experts (Seker and Zavadskas, 2017).

**Table 5:** Varying weights of the experts for robustness analysis in different scenarios.

Expert No.	Scenario 1	Scenario 2	Scenario 3	Scenario 4	Scenario 5
Expert 1	0.15	0.1	0.18	0.2	0.230
Expert 2	0.15	0.1	0.18	0.2	0.230
Expert 3	0.15	0.1	0.18	0.2	0.230
Expert 4	0.1	0.1	0.09	0.07	0.090
Expert 5	0.1	0.1	0.08	0.07	0.030
Expert 6	0.1	0.1	0.1	0.1	0.150
Expert 7	0.1	0.1	0.08	0.07	0.020
Expert 8	0.05	0.1	0.04	0.03	0.009
Expert 9	0.05	0.1	0.03	0.03	0.002
Expert 10	0.05	0.1	0.04	0.03	0.009

**Figure 2:**
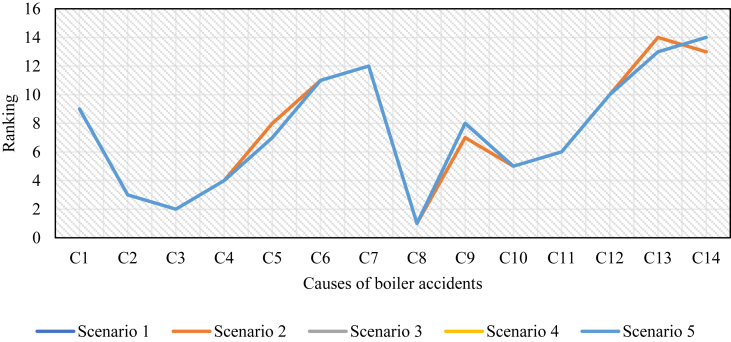
Robustness analysis for ranking the “causes of boiler accidents”.

For all these 5 scenarios, N-AHP has been carried out on the same data and the rankings for the different scenarios were obtained. Figure 2 represents the ranking of the “causes of boiler accidents” under different scenarios. From this figure, it can be seen that the ranking of the “causes of boiler accidents” remains almost stable though the weights of the experts have been varied. Therefore, it is obvious that the ranking of the causes of boiler accidents are not sensitive to the varying weights of the experts and hence, it indicates the higher robustness of the results of the current study.

## 5 Conclusion

This study has contributed to the safety science literature by proposing the N-AHP based methodological framework to identify, examine and prioritize the causes of boiler accidents in the RMG industry. The methodological framework and findings provide valuable insights to industrial managers and policymakers to decide on remedial actions that should be emphasized more with a view to minimizing the frequency of boiler accidents in an emerging economy like Bangladesh.

This study has some limitations. The proposed N-AHP model works on expert's feedback, as a result individual biases can occur. In addition, the findings of this study may be limited to the RMG industry or the country context. However, the proposed methodological framework can be generalized and applied to other industrial and country contexts. In the future, other multi-criteria decision making (MCDM) methods such as decision-making trial and evaluation laboratory (DEMATEL), or rough set-based AHP can be applied to validate the findings of this study.

## Declarations

### Author contribution statement

Md. Ashiqur Rahman, Chitra Lekha Karmaker, Tazim Ahmed, Md. Ikram Khan, A.K.M Monjur Morshed, Syed Mithun Ali: Conceived and designed the experiments; Performed the experiments; Analyzed and interpreted the data; Contributed reagents, materials, analysis tools or data; Wrote the paper.

### Funding statement

This research did not receive any specific grant from funding agencies in the public, commercial, or not-for-profit sectors.

### Data availability statement

Data included in article/supp. material/referenced in article.

### Declaration of interest's statement

The authors declare no conflict of interest.

### Additional information

No additional information is available for this paper.
