# Supplementary material for: Modelling the causes of boiler accidents: implications for economic and social sustainability at the workplace
Source: Heliyon. 2022 Jun 7;8(6):e09601. doi: 10.1016/j.heliyon.2022.e09601 (PMC9193911; doi:10.1016/j.heliyon.2022.e09601)
Supplement: Supplement file [file mmc1.docx]

**Appendix A**

Table A1: Brief description of the participants’ profile.

| Characteristics | | | *n* | % |
| --- | --- | --- | --- | --- |
| Participants (n=40) | Experience | Up to 7 years | 14 | 35% |
|  |  | 8 – 12 years | 20 | 50% |
|  |  | More than 12 years | 6 | 15% |
|  | Job position | Entry level manager | 12 | 30% |
|  |  | Mid-level manager | 24 | 60% |
|  |  | Senior manager | 4 | 10% |

Questionnaire provided for the identification of the causes of boiler accidents

1. Background information of the participant:

1. Name:
2. Name of the company:
3. Designation:
4. Years of experience:
5. Assigned responsibilities:

2. Where is the location of the factory?

3. What is the estimated capacity of the boiler?

4. What are the causes of boiler accidents?

**Appendix B**

Questionnaire provided for the evaluation of the causes of boiler accidents

1. Background information of the expert:

1. Name:
2. Name of the company:
3. Designation:
4. Years of experience:
5. Assigned responsibilities:

Table B1: Causes of boiler accidents.

| Causes | Code |
| --- | --- |
| Lack of regular boiler inspection and maintenance | C1 |
| Use of expired, non-registered, and non-certified boilers | C2 |
| Non-standard boiler operation | C3 |
| Faulty design of boiler | C4 |
| Improper control and safety system of boiler | C5 |
| Corrosion of critical parts of the boiler | C6 |
| Low quality material for boiler manufacturing | C7 |
| Lack of standard legislation | C8 |
| Lack of standard occupational health and safety practices | C9 |
| Shortage of skilled boiler operators | C10 |
| Inadequate regulatory authority | C11 |
| Failure of the safety valve | C12 |
| Poor water treatment | C13 |
| Conditions of the boiler room | C14 |

Table B2: Pair-wise comparison matrix to be filled.

| Causes | C1 | C2 | C3 | C4 | C5 | C6 | C7 | C8 | C9 | C10 | C11 | C12 | C13 | C14 |
| --- | --- | --- | --- | --- | --- | --- | --- | --- | --- | --- | --- | --- | --- | --- |
| C1 |  |  |  |  |  |  |  |  |  |  |  |  |  |  |
| C2 |  |  |  |  |  |  |  |  |  |  |  |  |  |  |
| C3 |  |  |  |  |  |  |  |  |  |  |  |  |  |  |
| C4 |  |  |  |  |  |  |  |  |  |  |  |  |  |  |
| C5 |  |  |  |  |  |  |  |  |  |  |  |  |  |  |
| C6 |  |  |  |  |  |  |  |  |  |  |  |  |  |  |
| C7 |  |  |  |  |  |  |  |  |  |  |  |  |  |  |
| C8 |  |  |  |  |  |  |  |  |  |  |  |  |  |  |
| C9 |  |  |  |  |  |  |  |  |  |  |  |  |  |  |
| C10 |  |  |  |  |  |  |  |  |  |  |  |  |  |  |
| C11 |  |  |  |  |  |  |  |  |  |  |  |  |  |  |
| C12 |  |  |  |  |  |  |  |  |  |  |  |  |  |  |
| C13 |  |  |  |  |  |  |  |  |  |  |  |  |  |  |
| C14 |  |  |  |  |  |  |  |  |  |  |  |  |  |  |

Table B3: Relative assessment of causes by Expert 1 using pair-wise comparison matrix in N-AHP.

| Causes | C1 | C2 | C3 | C4 | C5 | C6 | C7 | C8 | C9 | C10 | C11 | C12 | C13 | C14 |
| --- | --- | --- | --- | --- | --- | --- | --- | --- | --- | --- | --- | --- | --- | --- |
| C1 | 1 | 1/6 | 1/5 | 1/6 | 1/2 | 2 | 4 | 1/7 | 1/2 | 1/3 | 1/4 | 2 | 5 | 2 |
| C2 | 6 | 1 | 1/4 | 3 | 7 | 7 | 7 | 1/2 | 2 | 2 | 4 | 6 | 7 | 8 |
| C3 | 5 | 4 | 1 | 2 | 8 | 7 | 8 | 1/2 | 7 | 5 | 4 | 6 | 9 | 8 |
| C4 | 6 | 1/3 | 1/2 | 1 | 5 | 7 | 6 | 1/4 | 4 | 2 | 3 | 4 | 8 | 8 |
| C5 | 2 | 1/7 | 1/8 | 1/5 | 1 | 2 | 3 | 1/7 | 2 | 1/5 | 1/4 | 3 | 7 | 8 |
| C6 | 1/2 | 1/7 | 1/7 | 1/7 | 1/2 | 1 | 2 | 1/7 | 1/5 | 1/6 | 1/4 | 1/4 | 4 | 3 |
| C7 | 1/4 | 1/7 | 1/8 | 1/6 | 1/3 | 1/2 | 1 | 1/7 | 1/6 | 1/6 | 1/7 | 1/5 | 4 | 3 |
| C8 | 7 | 2 | 2 | 4 | 7 | 7 | 7 | 1 | 8 | 3 | 7 | 8 | 9 | 9 |
| C9 | 2 | 1/2 | 1/7 | 1/4 | 1/2 | 5 | 6 | 1/8 | 1 | 1/4 | 1/3 | 2 | 8 | 8 |
| C10 | 3 | 1/2 | 1/5 | 1/2 | 5 | 6 | 6 | 1/3 | 4 | 1 | 2 | 5 | 7 | 7 |
| C11 | 4 | 1/4 | 1/4 | 1/3 | 4 | 4 | 7 | 1/7 | 3 | 1/2 | 1 | 4 | 8 | 7 |
| C12 | 1/2 | 1/6 | 1/6 | 1/4 | 1/3 | 4 | 5 | 1/8 | 1/2 | 1/5 | 1/4 | 1 | 4 | 3 |
| C13 | 1/5 | 1/7 | 1/9 | 1/8 | 1/7 | 1/4 | 1/4 | 1/9 | 1/8 | 1/7 | 1/8 | 1/4 | 1 | 2 |
| C14 | 1/2 | 1/8 | 1/8 | 1/8 | 1/8 | 1/3 | 1/3 | 1/9 | 1/8 | 1/7 | 1/7 | 1/3 | 1/2 | 1 |

Table B4: Relative assessment of causes by Expert 2 using pair-wise comparison matrix in N-AHP.

| Causes | C1 | C2 | C3 | C4 | C5 | C6 | C7 | C8 | C9 | C10 | C11 | C12 | C13 | C14 |
| --- | --- | --- | --- | --- | --- | --- | --- | --- | --- | --- | --- | --- | --- | --- |
| C1 | 1 | 1/5 | 1/4 | 1/7 | 1/3 | 3 | 3 | 1/6 | 1/3 | 1/4 | 1/3 | 3 | 6 | 3 |
| C2 | 5 | 1 | 1/2 | 3 | 6 | 6 | 7 | 1/2 | 3 | 2 | 2 | 6 | 5 | 8 |
| C3 | 4 | 2 | 1 | 2 | 8 | 7 | 7 | 1/2 | 7 | 5 | 5 | 6 | 7 | 8 |
| C4 | 7 | 1/3 | 1/2 | 1 | 5 | 6 | 6 | 1/4 | 4 | 2 | 2 | 4 | 8 | 7 |
| C5 | 3 | 1/6 | 1/8 | 1/5 | 1 | 3 | 3 | 1/7 | 2 | 1/6 | 1/5 | 3 | 7 | 8 |
| C6 | 1/3 | 1/6 | 1/7 | 1/6 | 1/3 | 1 | 3 | 1/6 | 1/4 | 1/6 | 1/5 | 1/2 | 4 | 3 |
| C7 | 1/3 | 1/7 | 1/7 | 1/6 | 1/3 | 1/3 | 1 | 1/6 | 1/6 | 1/5 | 1/7 | 1/2 | 2 | 3 |
| C8 | 6 | 2 | 2 | 4 | 7 | 6 | 6 | 1 | 8 | 3 | 6 | 7 | 8 | 9 |
| C9 | 3 | 1/3 | 1/7 | 1/4 | 1/2 | 4 | 6 | 1/8 | 1 | 1/4 | 1/2 | 3 | 8 | 7 |
| C10 | 4 | 1/2 | 1/5 | 1/2 | 6 | 6 | 5 | 1/3 | 4 | 1 | 2 | 5 | 6 | 6 |
| C11 | 3 | 1/2 | 1/5 | 1/2 | 5 | 5 | 7 | 1/6 | 2 | 1/2 | 1 | 5 | 8 | 6 |
| C12 | 1/3 | 1/6 | 1/6 | 1/4 | 1/3 | 2 | 2 | 1/7 | 1/3 | 1/5 | 1/5 | 1 | 2 | 4 |
| C13 | 1/6 | 1/5 | 1/7 | 1/8 | 1/7 | 1/4 | 1/2 | 1/8 | 1/8 | 1/6 | 1/8 | 1/2 | 1 | 3 |
| C14 | 1/3 | 1/8 | 1/8 | 1/7 | 1/8 | 1/3 | 1/3 | 1/9 | 1/7 | 1/6 | 1/6 | 1/4 | 1/3 | 1 |

Table B5: Relative assessment of causes by Expert 3 using pair-wise comparison matrix in N-AHP.

| Causes | C1 | C2 | C3 | C4 | C5 | C6 | C7 | C8 | C9 | C10 | C11 | C12 | C13 | C14 |
| --- | --- | --- | --- | --- | --- | --- | --- | --- | --- | --- | --- | --- | --- | --- |
| C1 | 1 | 1/6 | 1/4 | 1/6 | 1/2 | 4 | 4 | 1/5 | 1/2 | 1/4 | 1/2 | 3 | 7 | 4 |
| C2 | 6 | 1 | 1/3 | 3 | 6 | 7 | 7 | 1/3 | 4 | 3 | 4 | 5 | 6 | 7 |
| C3 | 4 | 3 | 1 | 2 | 7 | 7 | 6 | 1/3 | 7 | 4 | 6 | 5 | 7 | 8 |
| C4 | 6 | 1/3 | 1/2 | 1 | 4 | 6 | 6 | 1/3 | 4 | 3 | 4 | 4 | 8 | 6 |
| C5 | 2 | 1/6 | 1/7 | 1/4 | 1 | 4 | 3 | 1/8 | 3 | 1/2 | 1/2 | 2 | 6 | 8 |
| C6 | 1/4 | 1/7 | 1/7 | 1/6 | 1/4 | 1 | 2 | 1/6 | 1/3 | 1/7 | 1/6 | 1/3 | 2 | 2 |
| C7 | 1/4 | 1/7 | 1/6 | 1/6 | 1/3 | 1/2 | 1 | 1/9 | 1/6 | 1/6 | 1/7 | 1/2 | 3 | 2 |
| C8 | 5 | 3 | 3 | 3 | 8 | 6 | 9 | 1 | 8 | 3 | 7 | 6 | 8 | 9 |
| C9 | 2 | 1/4 | 1/7 | 1/4 | 1/3 | 3 | 6 | 1/8 | 1 | 1/3 | 1/3 | 3 | 7 | 7 |
| C10 | 4 | 1/3 | 1/4 | 1/3 | 2 | 7 | 6 | 1/3 | 3 | 1 | 3 | 6 | 5 | 6 |
| C11 | 2 | 1/4 | 1/6 | 1/4 | 2 | 6 | 7 | 1/7 | 3 | 1/3 | 1 | 5 | 7 | 6 |
| C12 | 1/3 | 1/5 | 1/5 | 1/4 | 1/2 | 3 | 2 | 1/6 | 1/3 | 1/6 | 1/5 | 1 | 3 | 4 |
| C13 | 1/7 | 1/6 | 1/7 | 1/8 | 1/6 | 1/2 | 1/3 | 1/8 | 1/7 | 1/5 | 1/7 | 1/3 | 1 | 3 |
| C14 | 1/4 | 1/7 | 1/8 | 1/6 | 1/8 | 1/2 | 1/2 | 1/9 | 1/7 | 1/6 | 1/6 | 1/4 | 1/3 | 1 |

Table B6: Relative assessment of causes by Expert 4 using pair-wise comparison matrix in N-AHP.

| Causes | C1 | C2 | C3 | C4 | C5 | C6 | C7 | C8 | C9 | C10 | C11 | C12 | C13 | C14 |
| --- | --- | --- | --- | --- | --- | --- | --- | --- | --- | --- | --- | --- | --- | --- |
| C1 | 1 | 1/6 | 1/5 | 1/6 | 1/2 | 2 | 4 | 1/7 | 1/2 | 1/3 | 1/4 | 2 | 5 | 2 |
| C2 | 6 | 1 | 1/4 | 3 | 7 | 7 | 7 | 1/2 | 2 | 2 | 4 | 6 | 7 | 8 |
| C3 | 5 | 4 | 1 | 2 | 8 | 7 | 8 | 1/2 | 7 | 5 | 4 | 6 | 9 | 8 |
| C4 | 6 | 1/3 | 1/2 | 1 | 5 | 7 | 6 | 1/4 | 4 | 2 | 3 | 4 | 8 | 8 |
| C5 | 2 | 1/7 | 1/8 | 1/5 | 1 | 2 | 3 | 1/7 | 2 | 1/5 | 1/4 | 3 | 7 | 8 |
| C6 | 1/2 | 1/7 | 1/7 | 1/7 | 1/2 | 1 | 2 | 1/7 | 1/5 | 1/6 | 1/4 | 1/4 | 4 | 3 |
| C7 | 1/4 | 1/7 | 1/8 | 1/6 | 1/3 | 1/2 | 1 | 1/7 | 1/6 | 1/6 | 1/7 | 1/5 | 4 | 3 |
| C8 | 7 | 2 | 2 | 4 | 7 | 7 | 7 | 1 | 8 | 3 | 7 | 8 | 9 | 9 |
| C9 | 2 | 1/2 | 1/7 | 1/4 | 1/2 | 5 | 6 | 1/8 | 1 | 1/4 | 1/3 | 2 | 8 | 8 |
| C10 | 3 | 1/2 | 1/5 | 1/2 | 5 | 6 | 6 | 1/3 | 4 | 1 | 2 | 5 | 7 | 7 |
| C11 | 4 | 1/4 | 1/4 | 1/3 | 4 | 4 | 7 | 1/7 | 3 | 1/2 | 1 | 4 | 8 | 7 |
| C12 | 1/2 | 1/6 | 1/6 | 1/4 | 1/3 | 4 | 5 | 1/8 | 1/2 | 1/5 | 1/4 | 1 | 4 | 3 |
| C13 | 1/5 | 1/7 | 1/9 | 1/8 | 1/7 | 1/4 | 1/4 | 1/9 | 1/8 | 1/7 | 1/8 | 1/4 | 1 | 2 |
| C14 | 1/2 | 1/8 | 1/8 | 1/8 | 1/8 | 1/3 | 1/3 | 1/9 | 1/8 | 1/7 | 1/7 | 1/3 | 1/2 | 1 |

Table B7: Relative assessment of causes by Expert 5 using pair-wise comparison matrix in N-AHP.

| Causes | C1 | C2 | C3 | C4 | C5 | C6 | C7 | C8 | C9 | C10 | C11 | C12 | C13 | C14 |
| --- | --- | --- | --- | --- | --- | --- | --- | --- | --- | --- | --- | --- | --- | --- |
| C1 | 1 | 1/6 | 1/4 | 1/7 | 1/2 | 2 | 4 | 1/6 | 1/2 | 1/4 | 1/2 | 4 | 7 | 4 |
| C2 | 6 | 1 | 1/3 | 3 | 6 | 7 | 8 | 1/3 | 4 | 3 | 4 | 5 | 6 | 8 |
| C3 | 4 | 3 | 1 | 3 | 7 | 7 | 6 | 1/3 | 7 | 5 | 6 | 4 | 8 | 8 |
| C4 | 7 | 1/3 | 1/3 | 1 | 4 | 6 | 6 | 1/3 | 4 | 3 | 3 | 4 | 7 | 6 |
| C5 | 2 | 1/6 | 1/7 | 1/4 | 1 | 4 | 3 | 1/8 | 2 | 1/3 | 1/2 | 5 | 5 | 8 |
| C6 | 1/2 | 1/7 | 1/7 | 1/6 | 1/4 | 1 | 2 | 1/6 | 1/3 | 1/7 | 1/6 | 1/2 | 2 | 2 |
| C7 | 1/4 | 1/8 | 1/6 | 1/6 | 1/3 | 1/2 | 1 | 1/9 | 1/6 | 1/6 | 1/8 | 1/2 | 2 | 2 |
| C8 | 6 | 3 | 3 | 3 | 8 | 6 | 9 | 1 | 9 | 4 | 7 | 6 | 9 | 9 |
| C9 | 2 | 1/4 | 1/7 | 1/4 | 1/2 | 3 | 6 | 1/9 | 1 | 1/3 | 1/3 | 4 | 7 | 7 |
| C10 | 4 | 1/3 | 1/5 | 1/3 | 3 | 7 | 6 | 1/4 | 3 | 1 | 2 | 7 | 4 | 6 |
| C11 | 2 | 1/4 | 1/6 | 1/3 | 2 | 6 | 8 | 1/7 | 3 | 1/2 | 1 | 4 | 8 | 4 |
| C12 | 1/4 | 1/5 | 1/4 | 1/4 | 1/5 | 2 | 2 | 1/6 | 1/4 | 1/7 | 1/4 | 1 | 2 | 2 |
| C13 | 1/7 | 1/6 | 1/8 | 1/7 | 1/5 | 1/2 | 1/2 | 1/9 | 1/7 | 1/4 | 1/8 | 1/2 | 1 | 1/2 |
| C14 | 1/4 | 1/8 | 1/8 | 1/6 | 1/8 | 1/2 | 1/2 | 1/9 | 1/7 | 1/6 | 1/4 | 1/2 | 2 | 1 |

Table B8: Relative assessment of causes by Expert 6 using pair-wise comparison matrix in N-AHP.

| Causes | C1 | C2 | C3 | C4 | C5 | C6 | C7 | C8 | C9 | C10 | C11 | C12 | C13 | C14 |
| --- | --- | --- | --- | --- | --- | --- | --- | --- | --- | --- | --- | --- | --- | --- |
| C1 | 1 | 1/6 | 1/3 | 1/8 | 1/3 | 2 | 3 | 1/7 | 1/2 | 1/5 | 1/3 | 2 | 6 | 4 |
| C2 | 6 | 1 | 1/2 | 3 | 6 | 7 | 8 | 1/2 | 4 | 4 | 5 | 5 | 9 | 8 |
| C3 | 3 | 2 | 1 | 2 | 7 | 6 | 5 | 1/3 | 6 | 4 | 6 | 5 | 8 | 8 |
| C4 | 8 | 1/3 | 1/2 | 1 | 4 | 6 | 6 | 1/3 | 5 | 3 | 4 | 3 | 7 | 7 |
| C5 | 3 | 1/6 | 1/7 | 1/4 | 1 | 4 | 2 | 1/8 | 2 | 1/3 | 1/3 | 2 | 5 | 7 |
| C6 | 1/2 | 1/7 | 1/6 | 1/6 | 1/4 | 1 | 3 | 1/6 | 1/4 | 1/7 | 1/6 | 1/3 | 4 | 4 |
| C7 | 1/3 | 1/8 | 1/5 | 1/6 | 1/2 | 1/3 | 1 | 1/9 | 1/3 | 1/7 | 1/8 | 1/2 | 2 | 2 |
| C8 | 7 | 2 | 3 | 3 | 8 | 6 | 9 | 1 | 8 | 5 | 7 | 5 | 9 | 9 |
| C9 | 2 | 1/4 | 1/6 | 1/5 | 1/2 | 4 | 3 | 1/8 | 1 | 1/3 | 1/2 | 2 | 7 | 8 |
| C10 | 5 | 1/4 | 1/4 | 1/3 | 3 | 7 | 7 | 1/5 | 3 | 1 | 3 | 7 | 8 | 8 |
| C11 | 3 | 1/5 | 1/6 | 1/4 | 3 | 6 | 8 | 1/7 | 2 | 1/3 | 1 | 4 | 9 | 3 |
| C12 | 1/2 | 1/5 | 1/5 | 1/3 | 1/2 | 3 | 2 | 1/5 | 1/2 | 1/7 | 1/4 | 1 | 5 | 5 |
| C13 | 1/6 | 1/9 | 1/8 | 1/7 | 1/5 | 1/4 | 1/2 | 1/9 | 1/7 | 1/8 | 1/9 | 1/5 | 1 | 1/2 |
| C14 | 1/4 | 1/8 | 1/8 | 1/7 | 1/7 | 1/4 | 1/2 | 1/9 | 1/8 | 1/8 | 1/3 | 1/5 | 2 | 1 |

Table B9: Relative assessment of causes by Expert 7 using pair-wise comparison matrix in N-AHP.

| Causes | C1 | C2 | C3 | C4 | C5 | C6 | C7 | C8 | C9 | C10 | C11 | C12 | C13 | C14 |
| --- | --- | --- | --- | --- | --- | --- | --- | --- | --- | --- | --- | --- | --- | --- |
| C1 | 1 | 1/6 | 1/4 | 1/7 | 1/2 | 2 | 4 | 1/6 | 1/2 | 1/5 | 1/3 | 2 | 6 | 4 |
| C2 | 6 | 1 | 1/3 | 3 | 6 | 7 | 8 | 1/3 | 4 | 4 | 5 | 5 | 9 | 8 |
| C3 | 4 | 3 | 1 | 3 | 7 | 7 | 6 | 1/3 | 7 | 4 | 6 | 5 | 8 | 8 |
| C4 | 7 | 1/3 | 1/3 | 1 | 4 | 6 | 6 | 1/3 | 4 | 3 | 4 | 3 | 7 | 7 |
| C5 | 2 | 1/6 | 1/7 | 1/4 | 1 | 4 | 3 | 1/8 | 2 | 1/3 | 1/3 | 2 | 5 | 7 |
| C6 | 1/2 | 1/7 | 1/7 | 1/6 | 1/4 | 1 | 2 | 1/6 | 1/3 | 1/7 | 1/6 | 1/3 | 4 | 4 |
| C7 | 1/4 | 1/8 | 1/6 | 1/6 | 1/3 | 1/2 | 1 | 1/9 | 1/6 | 1/7 | 1/8 | 1/2 | 2 | 2 |
| C8 | 6 | 3 | 3 | 3 | 8 | 6 | 9 | 1 | 9 | 5 | 7 | 5 | 9 | 9 |
| C9 | 2 | 1/4 | 1/7 | 1/4 | 1/2 | 3 | 6 | 1/9 | 1 | 1/3 | 1/2 | 2 | 7 | 8 |
| C10 | 4 | 1/3 | 1/5 | 1/3 | 3 | 7 | 6 | 1/4 | 3 | 1 | 3 | 7 | 8 | 8 |
| C11 | 2 | 1/4 | 1/6 | 1/3 | 2 | 6 | 8 | 1/7 | 3 | 1/3 | 1 | 4 | 9 | 3 |
| C12 | 1/4 | 1/5 | 1/4 | 1/4 | 1/5 | 2 | 2 | 1/6 | 1/4 | 1/7 | 1/4 | 1 | 5 | 5 |
| C13 | 1/7 | 1/6 | 1/8 | 1/7 | 1/5 | 1/2 | 1/2 | 1/9 | 1/7 | 1/8 | 1/9 | 1/5 | 1 | 1/2 |
| C14 | 1/4 | 1/8 | 1/8 | 1/6 | 1/8 | 1/2 | 1/2 | 1/9 | 1/7 | 1/8 | 1/3 | 1/5 | 2 | 1 |

Table B10: Relative assessment of causes by Expert 8 using pair-wise comparison matrix in N-AHP.

| Causes | C1 | C2 | C3 | C4 | C5 | C6 | C7 | C8 | C9 | C10 | C11 | C12 | C13 | C14 |
| --- | --- | --- | --- | --- | --- | --- | --- | --- | --- | --- | --- | --- | --- | --- |
| C1 | 1 | 1/6 | 1/6 | 1/6 | 1/2 | 3 | 4 | 1/6 | 1/2 | 1/6 | 1/4 | 2 | 7 | 5 |
| C2 | 6 | 1 | 1/3 | 2 | 7 | 8 | 9 | 1/3 | 5 | 2 | 2 | 6 | 9 | 9 |
| C3 | 6 | 3 | 1 | 3 | 8 | 7 | 6 | 1/2 | 7 | 5 | 7 | 6 | 9 | 8 |
| C4 | 6 | 1/2 | 1/3 | 1 | 5 | 7 | 7 | 1/4 | 6 | 2 | 2 | 4 | 8 | 8 |
| C5 | 2 | 1/7 | 1/8 | 0.2 | 1 | 5 | 3 | 1/8 | 2 | 1/4 | 1/2 | 3 | 6 | 8 |
| C6 | 1/3 | 1/8 | 1/7 | 1/7 | 1/5 | 1 | 2 | 1/9 | 1/5 | 1/8 | 1/7 | 1/4 | 2 | 2 |
| C7 | 1/4 | 1/9 | 1/6 | 1/7 | 1/3 | 1/2 | 1 | 1/9 | 1/4 | 1/7 | 1/8 | 1/3 | 3 | 3 |
| C8 | 6 | 3 | 2 | 4 | 8 | 9 | 9 | 1 | 8 | 6 | 8 | 6 | 9 | 9 |
| C9 | 2 | 0.2 | 1/7 | 1/6 | 1/2 | 5 | 4 | 1/8 | 1 | 1/4 | 1/3 | 3 | 8 | 4 |
| C10 | 6 | 0.5 | 1/5 | 1/2 | 4 | 8 | 7 | 1/6 | 4 | 1 | 2 | 7 | 8 | 8 |
| C11 | 4 | 0.5 | 1/7 | 1/2 | 2 | 7 | 8 | 1/8 | 3 | 1/2 | 1 | 5 | 9 | 4 |
| C12 | 1/2 | 1/6 | 1/6 | 1/4 | 1/3 | 4 | 3 | 1/6 | 1/3 | 1/7 | 0.2 | 1 | 5 | 5 |
| C13 | 1/7 | 1/9 | 1/9 | 1/8 | 1/6 | 1/2 | 1/3 | 1/9 | 1/8 | 1/8 | 1/9 | 1/5 | 1 | 1/3 |
| C14 | 1/5 | 1/9 | 1/8 | 1/8 | 1/8 | 1/2 | 1/3 | 1/9 | 1/4 | 1/8 | 1/4 | 1/5 | 3 | 1 |

Table B11: Relative assessment of causes by Expert 9 using pair-wise comparison matrix in N-AHP

| Causes | C1 | C2 | C3 | C4 | C5 | C6 | C7 | C8 | C9 | C10 | C11 | C12 | C13 | C14 |
| --- | --- | --- | --- | --- | --- | --- | --- | --- | --- | --- | --- | --- | --- | --- |
| C1 | 1 | 1/6 | 1/3 | 1/8 | 1/3 | 2 | 3 | 1/7 | 1/2 | 1/5 | 1/3 | 2 | 7 | 4 |
| C2 | 6 | 1 | 1/2 | 3 | 6 | 7 | 8 | 1/2 | 4 | 4 | 5 | 5 | 6 | 8 |
| C3 | 3 | 2 | 1 | 2 | 7 | 6 | 5 | 1/3 | 6 | 4 | 6 | 5 | 8 | 8 |
| C4 | 8 | 1/3 | 1/2 | 1 | 4 | 6 | 6 | 1/3 | 5 | 3 | 4 | 3 | 7 | 6 |
| C5 | 3 | 1/6 | 1/7 | 1/4 | 1 | 4 | 2 | 1/8 | 2 | 1/3 | 1/3 | 2 | 5 | 8 |
| C6 | 1/2 | 1/7 | 1/6 | 1/6 | 1/4 | 1 | 3 | 1/6 | 1/4 | 1/7 | 1/6 | 1/3 | 2 | 2 |
| C7 | 1/3 | 1/8 | 1/5 | 1/6 | 1/2 | 1/3 | 1 | 1/9 | 1/3 | 1/7 | 1/8 | 1/2 | 2 | 2 |
| C8 | 7 | 2 | 3 | 3 | 8 | 6 | 9 | 1 | 8 | 5 | 7 | 5 | 9 | 9 |
| C9 | 2 | 1/4 | 1/6 | 1/5 | 1/2 | 4 | 3 | 1/8 | 1 | 1/3 | 1/2 | 2 | 7 | 7 |
| C10 | 5 | 1/4 | 1/4 | 1/3 | 3 | 7 | 7 | 1/5 | 3 | 1 | 3 | 7 | 4 | 6 |
| C11 | 3 | 1/5 | 1/6 | 1/4 | 3 | 6 | 8 | 1/7 | 2 | 1/3 | 1 | 4 | 8 | 4 |
| C12 | 1/2 | 1/5 | 1/5 | 1/3 | 1/2 | 3 | 2 | 1/5 | 1/2 | 1/7 | 1/4 | 1 | 2 | 2 |
| C13 | 1/6 | 1/9 | 1/8 | 1/7 | 1/5 | 1/4 | 1/2 | 1/9 | 1/7 | 1/8 | 1/9 | 1/5 | 1 | 1/2 |
| C14 | 1/4 | 1/8 | 1/8 | 1/7 | 1/7 | 1/4 | 1/2 | 1/9 | 1/8 | 1/8 | 1/3 | 1/5 | 2 | 1 |

Table B12: Relative assessment of causes by Expert 10 using pair-wise comparison matrix in N-AHP.

| Causes | C1 | C2 | C3 | C4 | C5 | C6 | C7 | C8 | C9 | C10 | C11 | C12 | C13 | C14 |
| --- | --- | --- | --- | --- | --- | --- | --- | --- | --- | --- | --- | --- | --- | --- |
| C1 | 1 | 1/6 | 1/4 | 1/6 | 1/2 | 4 | 4 | 1/5 | 1/2 | 1/4 | 1/2 | 3 | 7 | 4 |
| C2 | 6 | 1 | 1/3 | 3 | 6 | 7 | 7 | 1/3 | 4 | 3 | 4 | 5 | 6 | 7 |
| C3 | 4 | 3 | 1 | 2 | 7 | 7 | 6 | 1/3 | 7 | 4 | 6 | 5 | 7 | 8 |
| C4 | 6 | 1/3 | 1/2 | 1 | 4 | 6 | 6 | 1/3 | 4 | 3 | 4 | 4 | 8 | 6 |
| C5 | 2 | 1/6 | 1/7 | 1/4 | 1 | 4 | 3 | 1/8 | 3 | 1/2 | 1/2 | 2 | 6 | 8 |
| C6 | 1/4 | 1/7 | 1/7 | 1/6 | 1/4 | 1 | 2 | 1/6 | 1/3 | 1/7 | 1/6 | 1/3 | 2 | 2 |
| C7 | 1/4 | 1/7 | 1/6 | 1/6 | 1/3 | 1/2 | 1 | 1/9 | 1/6 | 1/6 | 1/7 | 1/2 | 3 | 2 |
| C8 | 5 | 3 | 3 | 3 | 8 | 6 | 9 | 1 | 8 | 3 | 7 | 6 | 8 | 9 |
| C9 | 2 | 1/4 | 1/7 | 1/4 | 1/3 | 3 | 6 | 1/8 | 1 | 1/3 | 1/3 | 3 | 7 | 7 |
| C10 | 4 | 1/3 | 1/4 | 1/3 | 2 | 7 | 6 | 1/3 | 3 | 1 | 3 | 6 | 5 | 6 |
| C11 | 2 | 1/4 | 1/6 | 1/4 | 2 | 6 | 7 | 1/7 | 3 | 1/3 | 1 | 5 | 7 | 6 |
| C12 | 1/3 | 1/5 | 1/5 | 1/4 | 1/2 | 3 | 2 | 1/6 | 1/3 | 1/6 | 1/5 | 1 | 3 | 4 |
| C13 | 1/7 | 1/6 | 1/7 | 1/8 | 1/6 | 1/2 | 1/3 | 1/8 | 1/7 | 1/5 | 1/7 | 1/3 | 1 | 3 |
| C14 | 1/4 | 1/7 | 1/8 | 1/6 | 1/8 | 1/2 | 1/2 | 1/9 | 1/7 | 1/6 | 1/6 | 1/4 | 1/3 | 1 |

Table B13. Aggregated neutrosophic pair-wise comparison matrix.

| Causes | C1 | C2 | C3 – C13 | C14 |
| --- | --- | --- | --- | --- |
| C1 | $<\left( 1,1,1,1 \right);0.85, 0.13, 0.15>$ | $<\left( 0.13, 0.15, 0.18, 0.21 \right);1, 0, 0>$ | … | $<\left( 2.60, 3.60, 5.15, 6.15 \right);0.56, 0.41, 0.44>$ |
| C2 | $<\left( 4.75, 5.75, 6.75, 7.75 \right);0.85, 0.13, 0.15>$ | $<\left( 1, 1, 1, 1 \right);0.5, 0.5, 0.5>$ | … | $<\left( 7.00, 7.90, 8.80, 9.00 \right);1, 0, 0>$ |
| C3 | $<\left( 3.4, 4.4, 5.6, 6.6 \right);0.70, 0.26, 0.30>$ | $<\left( 1.85, 2.85, 5.10, 6.10 \right);0.41, 0.61, 0.59>$ | … | $<\left( 7.00, 8.00, 9.00, 9.00 \right);0.85, 0.10, 0.15>$ |
| C4 | $<\left( 5.6, 6.6, 7.6, 8.45 \right);0.86, 0.13, 0.14>$ | $<\left( 0.15, 0.18, 0.36, 0.58 \right);1, 0, 0>$ | … | $<\left( 6.15, 7.15, 8.15, 8.75 \right);0.88, 0.10, 0.12>$ |
| C5 | $<\left( 1.4, 2.4, 4.8, 5.8 \right);0.36, 0.69, 0.64>$ | $<\left( 0.13, 0.14, 0.16, 0.19 \right);1, 0, 0>$ | … | $<\left( 6.80, 7.80, 8.80, 9.00 \right);0.86, 0.10, 0.14>$ |
| C6 | $<\left( 0.18, 0.22, 0.40, 0.71 \right);1, 0, 0>$ | $<\left( 0.11, 0.13, 0.14, 0.17 \right);1, 0, 0>$ | … | $<\left( 1.65, 2.65, 4.85, 5.85 \right);0.40, 0.62, 0.60>$ |
| C7 | $<\left( 0.44, 0.57, 0.75, 0.95 \right);1, 0, 0>$ | $<\left( 0.11, 0.12, 0.13, 0.16 \right);1, 0, 0>$ | … | $<\left( 1.45, 2.45, 4.90, 5.90 \right);1, 0, 0>$ |
| C8 | $<\left( 5.05, 6.05, 7.05, 8.05 \right);0.89, 0.11, 0.11>$ | $<\left( 1.50, 2.50, 5.00, 6.00 \right);0.35, 0.70, 0.65>$ | … | $<\left( 9.00, 9.00, 9.00, 9.00 \right);1, 0, 0>$ |
| C9 | $<\left( 1.20, 2.20, 4.40, 5.40 \right);0.38, 0.67, 0.62>$ | $<\left( 0.17, 0.20, 0.32, 0.52 \right);1, 0, 0>$ | … | $<\left( 6.05, 7.05, 8.05, 8.70 \right);0.87, 0.11, 0.13>$ |
| C10 | $<\left( 3.25, 4.25, 5.65, 6.65 \right);0.67, 0.30, 0.33>$ | $<\left( 0.17, 0.21, 0.40, 0.72 \right);1, 0, 0>$ | … | $<\left( 5.95, 6.95, 7.95, 8.65 \right);0.89, 0.10, 0.11>$ |
| C11 | $<\left( 1.9, 2.9, 4.9, 5.9 \right);0.44, 0.56, 0.56>$ | $<\left( 0.17, 0.21, 0.31, 0.49 \right);1, 0, 0>$ | … | $<\left( 4.40, 5.40, 6.70, 7.70 \right);0.81, 0.19, 0.19>$ |
| C12 | $<\left( 0.17, 0.21, 0.39, 0.69 \right);1, 0, 0>$ | $<\left( 0.14, 0.16, 0.19, 0.23 \right);1, 0, 0>$ | … | $<\left( 2.75, 3.75, 5.25, 6.25 \right);0.62, 0.34, 0.38>$ |
| C13 | $<\left( 0.12, 0.14, 0.16, 0.19 \right);1, 0, 0>$ | $<\left( 0.12, 0.14, 0.16, 0.19 \right);1, 0, 0>$ | … | $<\left( 1.12, 1.74, 3.47, 4.23 \right);1, 0, 0>$ |
| C14 | $<\left( 0.17, 0.20, 0.32, 0.52 \right);1, 0, 0>$ | $<\left( 0.11, 0.11, 0.13, 0.14 \right);1, 0, 0>$ | … | $<\left( 1, 1, 1, 1 \right);0.5, 0.5, 0.5>$ |

Table B14. Neutrosophic synthetic value matrix.

| Causes | Synthetic value (S_i_) |
| --- | --- |
| C1 | $<\left( 0.02, 0.03, 0.06, 0.10 \right);1, 0, 0>$ |
| C2 | $<\left( 0.07, 0.10, 0.16, 0.22 \right);1, 0, 0>$ |
| C3 | $<\left( 0.08, 0.11, 0.18, 0.24 \right);1, 0, 0>$ |
| C4 | $<\left( 0.06, 0.09, 0.14, 0.20 \right);1, 0, 0>$ |
| C5 | $<\left( 0.03, 0.05, 0.09, 0.13 \right);1, 0, 0>$ |
| C6 | $<\left( 0.01, 0.02, 0.04, 0.05 \right);1, 0, 0>$ |
| C7 | $<\left( 0.01, 0.01, 0.03, 0.04 \right);1, 0, 0>$ |
| C8 | $<\left( 0.10, 0.13, 0.20, 0.27 \right);1, 0, 0>$ |
| C9 | $<\left( 0.04, 0.05, 0.09, 0.12 \right);1, 0, 0>$ |
| C10 | $<\left( 0.05, 0.08, 0.13, 0.18 \right);1, 0, 0>$ |
| C11 | $<\left( 0.05, 0.07, 0.11, 0.16 \right);1, 0, 0>$ |
| C12 | $<\left( 0.02, 0.02, 0.05, 0.07 \right);1, 0, 0>$ |
| C13 | $<\left( 0.01, 0.01, 0.01, 0.02 \right);1, 0, 0>$ |
| C14 | $<\left( 0.00, 0.01, 0.01, 0.02 \right);1, 0, 0>$ |

Table B15. Final importance weights in single valued trapezoidal neutrosophic numbers (SVTNNs).

| Causes | Weight (W_i_) |
| --- | --- |
| C1 | $<\left( 0.01, 0.03, 0.08, 0.17 \right);1, 0, 0>$ |
| C2 | $<\left( 0.04, 0.08, 0.20, 0.40 \right);1, 0, 0>$ |
| C3 | $<\left( 0.05, 0.09, 0.23, 0.44 \right);1, 0, 0>$ |
| C4 | $<\left( 0.03, 0.07, 0.18, 0.36 \right);1, 0, 0>$ |
| C5 | $<\left( 0.02, 0.04, 0.12, 0.23 \right);1, 0, 0>$ |
| C6 | $<\left( 0.01, 0.01, 0.05, 0.10 \right);1, 0, 0>$ |
| C7 | $<\left( 0.00, 0.01, 0.04, 0.08 \right);1, 0, 0>$ |
| C8 | $<\left( 0.05, 0.10, 0.26, 0.49 \right);1, 0, 0>$ |
| C9 | $<\left( 0.02, 0.04, 0.11, 0.22 \right);1, 0, 0>$ |
| C10 | $<\left( 0.03, 0.06, 0.16, 0.32 \right);1, 0, 0>$ |
| C11 | $<\left( 0.03, 0.05, 0.14, 0.29 \right);1, 0, 0>$ |
| C12 | $<\left( 0.01, 0.02, 0.06, 0.13 \right);1, 0, 0>$ |
| C13 | $<\left( 0.00, 0.01, 0.02, 0.04 \right);1, 0, 0>$ |
| C14 | $<\left( 0.00, 0.00, 0.01, 0.03 \right);1, 0, 0>$ |
